# A combination of tyrosine kinase inhibitors, crizotinib and dasatinib for the treatment of glioblastoma multiforme

**DOI:** 10.18632/oncotarget.5698

**Published:** 2015-10-16

**Authors:** Hayley Nehoff, Neha N. Parayath, Melanie J. McConnell, Sebastien Taurin, Khaled Greish

**Affiliations:** ^1^ Department of Pharmacology and Toxicology, University of Otago, Dunedin, New Zealand; ^2^ School of Biological Sciences, Victoria University of Wellington, Wellington, New Zealand

**Keywords:** glioblastoma multiforme, tyrosine kinase inhibitors, invasion, Met, SRC

## Abstract

Glioblastoma multiforme (GBM) is the most common and aggressive primary brain tumor. Despite the advances in surgery, radiotherapy and chemotherapy, patient survival averages only 14.6 months. In most GBM tumors, tyrosine kinases show increased activity and/or expression and actively contribute to the development, recurrence and onset of treatment resistance; making their inhibition an appealing therapeutic strategy. We compared the cytotoxicity of 12 tyrosine kinase inhibitors *in vitro*. A combination of crizotinib and dasatinib emerged as the most cytotoxic across established and primary human GBM cell lines. The combination treatment induced apoptotic cell death and polyploidy. Furthermore, the combination treatment led to the altered expression and localization of several tyrosine kinase receptors such as Met and EGFR and downstream effectors as such as SRC. Furthermore, the combination treatment reduced the migration and invasion of GBM cells and prevented endothelial cell tube formation *in vitro*. Overall, our study demonstrated the broad specificity of a combination of crizotinib and dasatinib across multiple GBM cell lines. These findings provide insight into the development of alternative therapy for the treatment of GBM.

## INTRODUCTION

Glioblastoma multiforme (GBM), a grade IV astrocytoma, is the most frequent and aggressive malignant primary brain tumor with a 5-year survival rate of 5% [1]. GBM is principally idiopathic, although several genetic mutations, epigenetic variations, viral infections and environmental factors have been identified as potential risk factors [2–5]. The standard treatment for GBM involves surgical resection and radiotherapy with concomitant and adjuvant temozolomide administration [6]. Despite the three-pronged treatment strategy, relapse is universal. The limited efficacy of the current treatments is partially a consequence of the heterogeneity of the tumour cell population. GBM is classified into four subtypes, the classical, mesenchymal, proneural and neural subtypes all characterised by distinct transcriptional profiles [7]. The subtypes are associated with the amplification and/or mutation of several receptor tyrosine kinases (RTKs) such as the hepatocyte growth factor receptor (Met), the platelet-derived growth factor receptor (PDGFR)-α and epidermal growth factor receptor (EGFR) in the mesenchymal, proneural and classical subtypes, respectively [7]. Furthermore, recent studies have also confirmed tumours harboring more than one subtype [8, 9].

The implication of RTKs in the development, recurrence and treatment resistance of GBM [10] has led to small molecule tyrosine kinase inhibitors (TKIs) emerging as new treatment options. We assessed 12 TKIs inhibiting various kinases associated with the development and progression of GBM. Dasatinib and crizotinib were the most potent and reduced cell viability in a panel of GBM cell lines. Dasatinib is a broad spectrum inhibitor of SRC family kinases such as the non-receptor tyrosine kinases SRC, Fyn and Lyn [11]. SRC is frequently constitutively activated in GBM cell lines and patient tumors [12] and contributes to the invasive potential of GBM *in vivo* [13]. Crizotinib inhibits the Met, ROS1 and anaplastic lymphoma kinase (ALK) RTKs. Met is expressed in all GBM cell lines [14], up to 72% of primary GBM tumors [15], promotes hypoxia driven invasion and the development of the pseudopalisades that are characteristic of GBM [16–18]. Furthermore, Met is also associated with the aquired resistance to cetuximab, a monoclonal antibody targeting EGFR [19]. ALK is poorly characterised in GBM but a number of reports suggest a role in the increased proliferation of GBM cells [20, 21].

In the current study, we demonstrate that a combination of dasatinib and crizotinib suppressed the viability of four established and two primary GBM cell lines. The combination also reduced the viability of GBM tumour spheroids. Moreover, our data indicates that the combination suppressed the activity and expression of Met, SRC and their downstream effectors. The combination synergistically increased apoptosis and abolished migration and invasion of the GBM cells and prevent neo-angiogenesis. Together, our results support the efficacy of the combination of two TKIs, dasatinib and crizotinib, for the treatment of GBM by targeting different oncogenic signaling pathways.

## RESULTS

### TKIs reduce GBM cell viability *in vitro*

**Figure 1:**
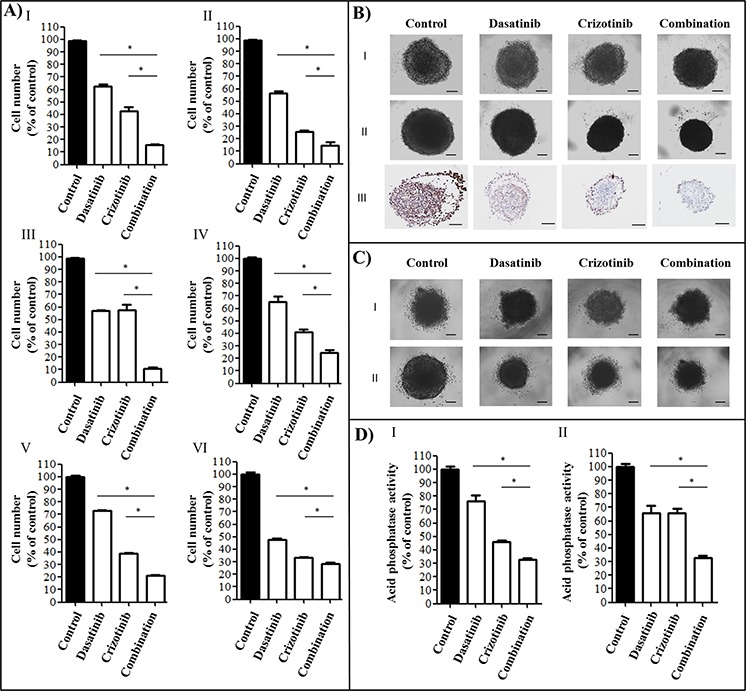
Cytotoxicity of a combination of dasatinib and crizotinib on GBM cell lines and GBM tumor spheroids **A.** Cell number was measured following 72 h of treatment at concentrations of 0.2 μM dasatinib and 4 μM crizotinib using an SRB assay in I: U87, II: LN-18, III: U373, IV: A172, V: NZG1003 and VI: NZG0906 cells. Data are expressed as the mean ± SEM. Experiments were conducted in triplicate and repeated independently three times. **B.** U87 spheroids I: before treatment, II: following 4 days treatment and III: Ki67 staining of spheroids treated for 4 days. Experiments were conducted in sextuplicate and repeated independently three times, representative images are shown. **C.** NZG1003 spheroids I: before treatment and II: following 4 days treatment. Experiments were conducted in sextuplicate and repeated independently three times, representative images are shown. **D.** Acid phosphatase assay of I: U87 and II: NZG1003 spheroids following 4 days of treatment. Data are expressed as the mean ± SEM. Experiments were conducted in sextuplicate and repeated independently three times. Scale bars denote 100 μm, * denotes *p* ≤ 0.05 as determined by an ANOVA with a Bonferroni post-hoc test.

We assessed the effect of several TKIs including sorafenib, nilotinib, sunitinib, imatinib, gefitinib, lapatinib, PD-173074, selumetinib, tofacitinib, pazopanib, dasatinib and crizotinib on the cellular viability of four established GBM cell lines (Table 1). The tyrosine kinases targeted were demonstrated to have an increased activity and/or expression in GBM and contribute to the progression, recurrence and treatment resistance of these tumors. The combination of dasatinib and crizotinib was the most cytotoxic across all the cell lines. We optimized the concentrations for both drugs to obtain a potent effect across all etablished and primary GBM cell lines used in this study and proceeded with 0.2 μM of dasatinib and 4 μM of crizotinib. Dasatinib and crizotinib treatment reduced the number of cells between 35 to 52% and 45 to 75%, respectively, across all cell lines over a 72 h period (Figure 1). Combination of crizotinib and dasatinib was more cytotoxic compared to the single treatments and decreased the number of cells by 71 to 90% (Figure 1A).

**Table 1:** The efficacy of TKIs against a panel of GBM cell lines

Drug	Targets	IC_50_ (μM)			
		U87	LN-18	U373	A172
Sorafenib	VEGFR-2 VEGFR-3 KIT PDGFR-β	7.3	4.9	8.0	6.2
Nilotinib	ABL1-2 PDGFR KIT BCR-ABL	12.5	14.9	>15	10.2
Sunitinib	VEGFR-1-2 VEGFR-3 KIT PDGFR	6.0	5.5	>15	12.0
Imatinib	ABL1-2 PDGFR KIT	>15	>15	>15	>15
Gefitinib	EGFR	>15	12.2	>15	>15
Lapatinib	EGFR ERBB2	12.5	7.0	12.6	12.4
Dasatinib	ABL1-2 PDGFR KIT SRC	0.31	0.25	0.5	2.35
PD-173074	FGFR-1	11.6	6.18	13.8	0.95
Selumetinib	MEK	14.75	>15	11.9	>15
Crizotinib	Met ALK	2.17	1.57	3.3	3.0
Tofacitinib	JAK3	>15	>15	>15	>15
Pazopanib	VEGFR-1-2 VEGFR-3 PDGFR KIT	>15	>15	>15	>15

### Cytotoxicity of the combination using GBM tumor spheroid models

The established GBM cell line U87 and the primary GBM cell line NZG1003 both form stable tumor spheroids, a three-dimensional culture that mimics some aspects of the *in vivo* tumor organization and often better recapitulates the response of the tumor to the drug. The spheroids were grown for 4 days and photographed before being treated with dasatinib, crizotinib or combination for 4 days (Figure 1B and 1C). At the end of the treatment period, spheroids were photographed and viability of the cells measured via an acid phosphatase activity assay (Figure 1DI-II). The combination was consistently more cytotoxic than the single treatments and decreased the viability of the tumor spheroids by nearly 70%. Furthermore, using the U87 spheroids, we measured the effect of treatment on cell proliferation using an antibody directed against Ki67, a cellular marker of proliferation (Figure 1BIII). The control spheroid exhibited an intense Ki67 staining on the surface of the spheroid. Treatment with dasatinib reduces Ki67 expression but has no effect on the spheroid size despite a reduction of the cell number by nearly 20% (Figure 1DI). The treatment with crizotinib decreases cell proliferation while the combination limited Ki67 expression to a small number of cells at the periphery of the tumor spheroid (Figure 1BIII).

### Cell signaling in response to treatment

**Figure 2:**
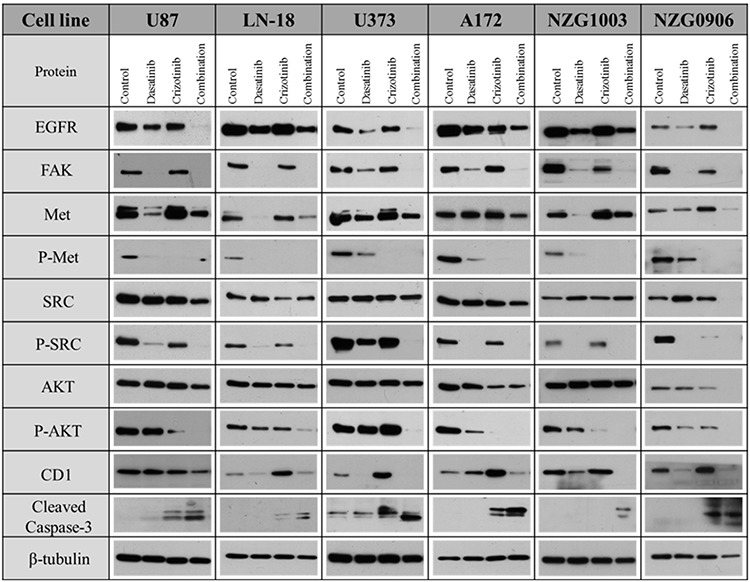
Combination of dasatinib and crizotinib decreases the activity and/or expression of Met, SRC and related proteins GBM established and primary cell lines were treated with dasatinib 0.2 μM, crizotinib 4 μM or their combination for 48 h. Total lysates were analyzed by western blotting with antibodies as indicated.

We then tested the effect of the combination treatment on the expression of proteins associated with cell proliferation, survival and invasion. The combination decreased EGFR expression in LN-18, A172 and NZG1003 cells while abolishing it in U87, U373 and NZG0906 cells. Furthermore, the combination abolishes the expression of focal adhesion kinase (FAK), a protein involved in the migration and invasion of cancer cells. Dasatinib was also highly effective in the suppression of FAK while crizotinib treatment slightly reduced its expression only in the two primary cell lines. The phosphorylation of Met, the RTK targeted by crizotinib, was significantly decreased by dasatinib treatment in U87, LN-18, U373 and NZG1003 cells, but not in A172 or NZG0906 cells while crizotinib increased Met expression in all cell lines. We then considered the effect of combination treatment on the downstream effectors of these kinases. In our study, the phosphorylation of SRC is abolished in all cell lines while the expression of total SRC is not consistently altered following dasatinib treatment (Figure 2). Treatment with crizotinib did not affect the expression of SRC but reduced its phosphorylation. The combination completely suppressed SRC phosphorylation in all cell lines (Figure 2). AKT is a key signal transduction pathway found to be constitutively active in multiple GBM cell lines and tumors. The combination completely abolishes AKT phosphorylation in all cell lines but total AKT expression was only abolished in combination treated NZG0906 cells. We also evaluated the effect of treatment on cyclin D1 (CD1) expression. Dasatinib is a potent cytostatic agent and reduced CD1 expression in all cell lines but U87 while crizotinib increased CD1 expression in all cell lines but U87. The combination treatment heavily reduced the CD1 expression in all cell lines relative to crizotinib treatment. Finally, we demonstrated that the activation of the apoptotic effector caspase-3 was increased in all four cell lines following crizotinib treatment and more strongly with combination treatment (Figure 2).

### Subcellular localization of tyrosine kinases

**Figure 3:**
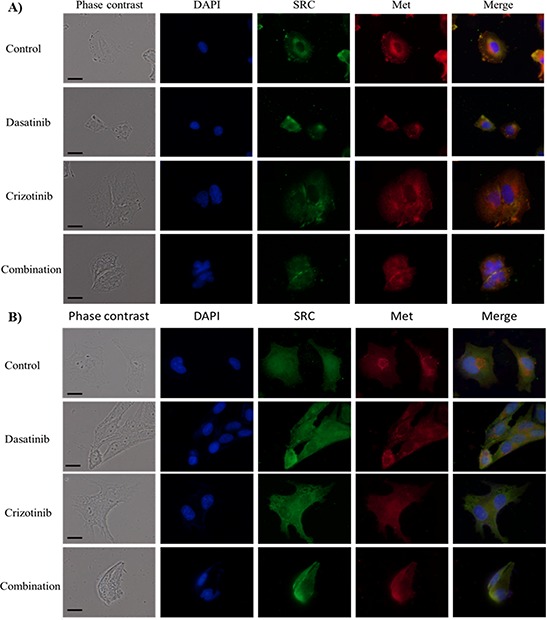
Immunocytochemistry of SRC and Met localization Cellular localization of SRC and Met following treatment for 48 h with dasatinib, crizotinib or combination in **A.** LN-18 and **B.** U373 cells. Scale bar denotes 10 μm. Nuclei were visualized by DAPI while SRC and Met were labeled with a fluorescein and Texas-red conjugated secondary antibodies, respectively.

We examined the effect of the different treatments on the intracellular localization of SRC and Met in two established cell lines, LN-18 and U373 cells. In LN-18 cells, SRC is abundant at the periphery of the nucleus and shows strong accumulation in the lamellipodia. Treatment with dasatinib, crizotinib or combination abolishes perinuclear and lamellipodial SRC localization and disperses SRC within the cytoplasm (Figure 3A). In U373 cells, SRC expression in the nucleus is reduced by the treatment with crizotinib, dasatinib and combination (Figure 3B). Met localizes primarily on the plasma membrane but also in the perinuclear region in both cell lines while in LN-18 cells, Met co-localizes with SRC. Treatment with crizotinib or combination reduces the membrane and perinuclear localization of Met, resulting in a diffuse cytoplasmic staining. Dasatinib also decreases the perinuclear localization of Met in LN-18 and U373 cells. Moreover, both treatments induce significant changes in the cellular morphology. SRC is known to influence the activity of multiple ion transporters [22] and treatment with dasatinib reduced the cell area by 50% in both cell lines. Met regulates cell cycle transition in the G_1_/S and G_2_/M phases of the cell cycle [23] and crizotinib treatment increased cell area by nearly 2.5- and 1.3-fold in LN-18 and U373 cells, respectively. In addition, crizotinib treatment promoted polynucleation in both cell lines. The combination treatment decreases the cell volume and polynucleation relative to crizotinib treatment.

### Mode of cell death induced by crizotinib, dasatinib and their combination

**Figure 4:**
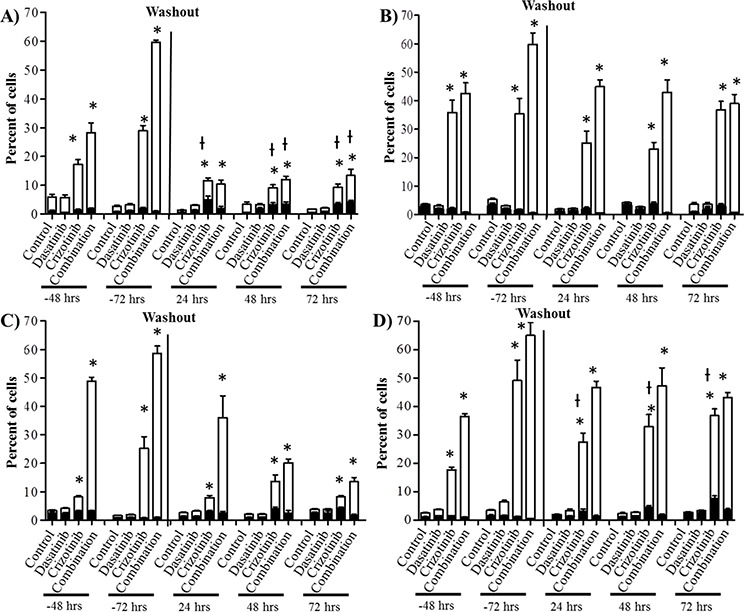
Combination treatment promotes apoptosis in GBM cell lines Cells were treated with dasatinib 0.2 μM, crizotinib 4 μM or their combination for up to 72 h followed by a washout period of up to 72 h, cell death was assessed at 48 and 72 h and every 24 h during the washout period. Bars denote necrotic; ■ (PI stained) and apoptotic; □ (Annexin V stained). **A.** U87, **B.** LN-18, **C.** U373, and **D.** A172 cells. Experiments were conducted in triplicate and independently repeated three times, *p* ≤ 0.05 relative to control for annexin V (*) and PI (ƚ).

We previously demonstrated by western blot that crizotinib and combination treatment resulted in the activation of caspase-3 (Figure 2). We further confirmed that the mode of cell death was via apoptosis using flow cytometry to measure early apoptotic events using annexin-V and PI staining as markers of the apoptotic and necrotic processes respectively. The concentration of dasatinib used in these experiments did not promote necrosis or apoptosis in any of the cell lines at any time point (Figure 4). The reduction in cell number observed with the SRB (Figure 1A), reduction in Ki67 staining of spheroids (Figure 1 BIII), lack of caspase-3 activation (Figure 2) and lack of apoptosis or necrosis induction (Figure 4) suggests that the mechanism of action of dasatinib is cytostatic. Conversely, crizotinib treatment induces apoptosis but not necrosis in all four established GBM cell lines (Figure 4). Following combination treatment, a synergistic increase in apoptosis was observed after treatment for 72 h in all cell lines (Figure 4). For example, in LN-18 cells, the percentage of apoptotic cells is 1.3, 33.9 and 59.3% following dasatinib, crizotinib and combination treatments respectively.

To determine if the effects triggered by dasatinib and crizotinib are transient or associated with prolonged changes, we removed the drug containing media, replaced it with fresh growth media and measured the apoptosis over the subsequent 72 h. The maintenance of apoptosis was heavily influenced by the cell line (Figure 4). In U87 cells, the number of apoptotic cells following combination treatment decreased rapidly to 8.4% 24 h after washout relative to 58.5% following 72 h of treatment. Importantly, the synergistic effect observed by the combination was lost and the apoptosis was comparable to the crizotinib treatment. Conversely, in LN-18, U373 and A172 cells, the synergistic increase of apoptosis triggered by the combination treatment was sustained over the 72 h. These results further emphasize the relevance of the combination relative to single-agent treatment by prolonging the apoptotic effect in most cell lines and permanently altering the ability of the cells to recover from treatment in susceptible GBM cell lines.

### Cell cycle

**Figure 5:**
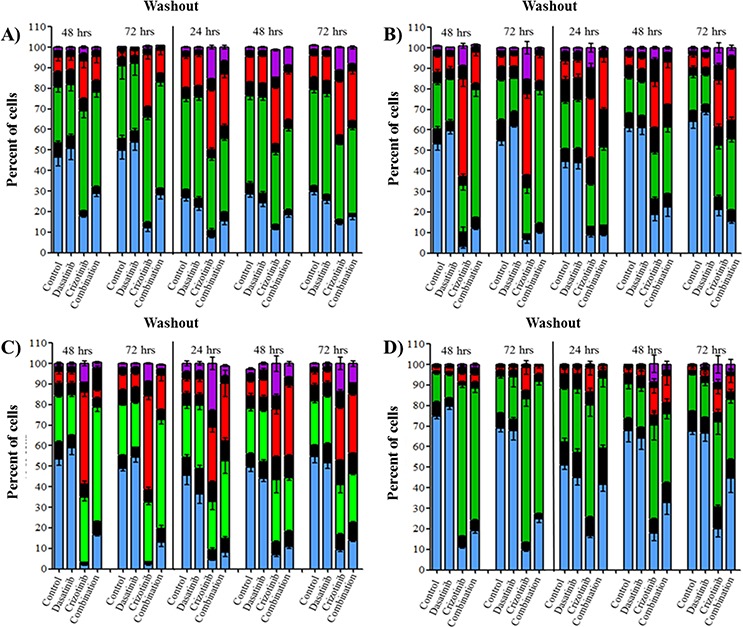
Combination treatment triggered cell cycle arrest in G2/M phase and increased occurrence of polyploid cells Cells were treated with dasatinib 0.2 μM, crizotinib 4 μM or their combination for up to 72 h followed by a washout period of up to 72 h, cell cycle was assessed at 48 and 72 h and every 24 h during the washout period. Bars denote cells with 1X DNA 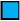, 2X DNA 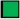, 4X DNA 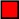, 8X DNA 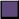 and the transition phases between the respective DNA content ■. **A.** U87, **B.** LN-18, **C.** U373, and **D.** A172 cells. Data are expressed as the mean ± SEM, experiments were conducted in triplicate and repeated independently three times. Statistical analysis is available in Supplementary Table S1–S4.

As shown previously, crizotinib treatment triggered polynucleation (Figure 3) while dasatinib treatment was sufficient to reduce cell number without inducing cell death (Figure 1, Figure 2 and Figure 4), suggesting that both drugs are able to alter DNA synthesis and cell cycle progression. Cell cycle analysis showed that dasatinib treatment transiently promotes accumulation of the cells in G1 phase of the cell cycle (1X DNA content) in all cell lines after 48 h treatment (Figure 5). Following treatment with crizotinib for 48 and 72 h, a significant number of cells accumulated in the G_2_/M phase (2X DNA content) of the cell cycle (*p* ≤ 0.01) (Supplementary Tables S1–S4). However, even though this cell population failed to complete mitosis, they were able to re-enter the cell cycle and cause the formation of tetraploid (4X DNA content) and octoploid (8X DNA content) cells. The effect of crizotinib on G_2_/M accumulation and polyploidy were consistent after 48 and 72 h incubation but differs in intensity across all cell lines. LN-18 and U373 showed a rapid increase of tetraploid (4X DNA content) and octoploid (8X DNA content) cell populations while U87 and A172 are mainly characterized by G_2_/M arrest (Figure 5). The combination treatment also increased the quantity of the polyploid cell population relative to control cells but significantly reduces the proportion of tetraploid and octoploid cells relative to crizotinib treatment. For example, in LN-18 cells, the number of tetraploid and octoploid cells following crizotinib treatment was 39.3 and 15.6% while the in the combination the number were reduced to 12.7 and 1.2%, respectively. Following washout, the polyploid cell population induced by crizotinib treatment is maintained while, in combination treated cells, the number of tetraploid and octoploid cells increased over the washout period. The presentation of polyploid cells (Figure 5) does not closely correlate with apoptosis (Figure 4) demonstrating that dysregulation of mitosis is not the determining factor for the induction of apoptotic cell death.

### Crizotinib treatment disrupts correct mitotic spindle formation

**Figure 6:**
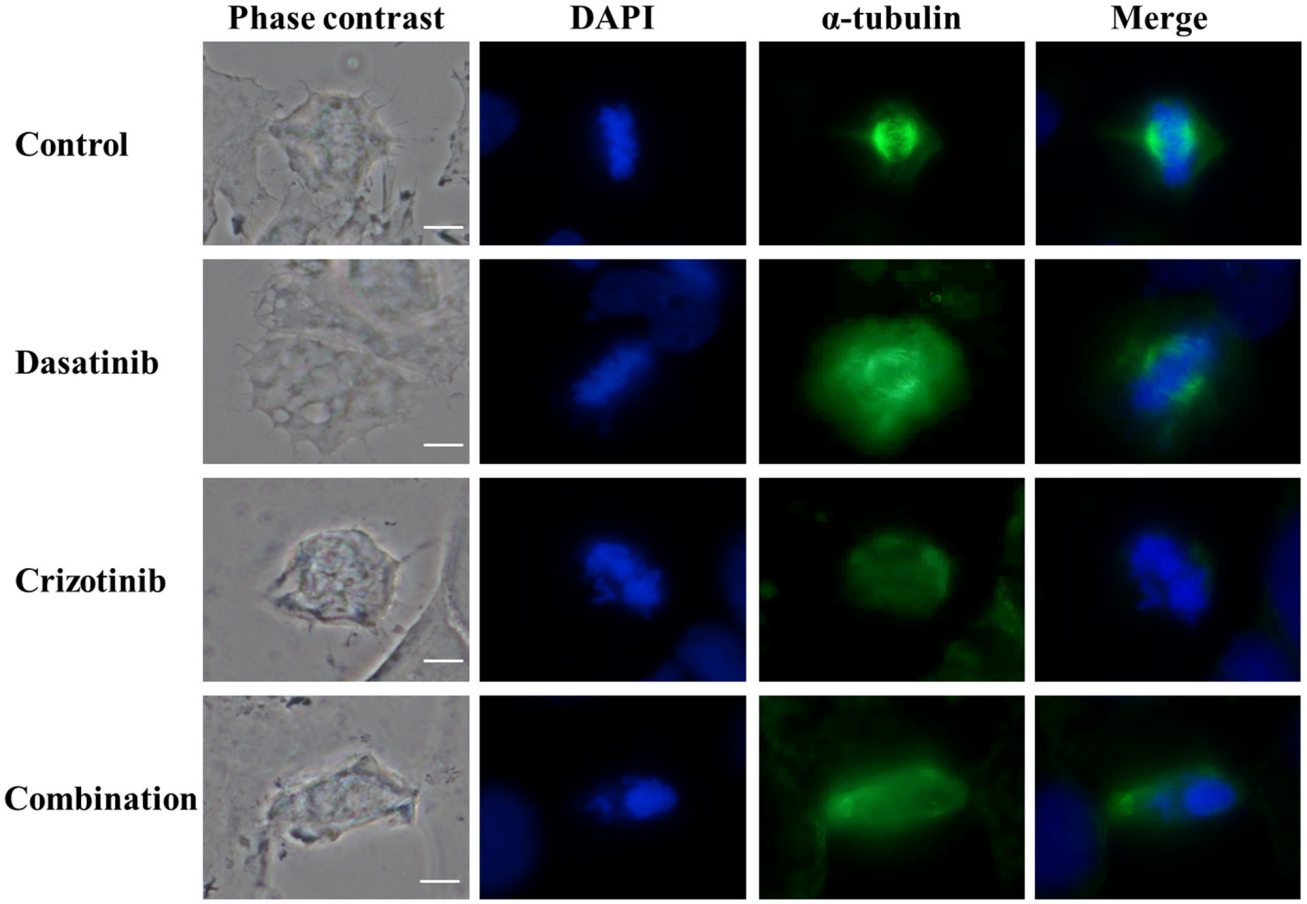
Combination of dasatinib and crizotinib impedes the formation of the α-tubulin-labeled mitotic spindle LN-18 cells were treated with dasatinib 0.2 μM, crizotinib 4 μM or their combination for 48 h. Mitotic spindle were visualized using α-tubulin antibody conjugated to a fluorescein-labeled secondary antibody. Nuclei were stained by DAPI. The scale bar denotes 10 μm.

We previously observed that following crizotinib or combination treatment, a number of cells had more than one nucleus (Figure 3) and had up to 8-fold the normal DNA content (Figure 5). Polynucleation and polyploidy is often a consequence of dysfunctional formation of the mitotic apparatus. The mitotic spindles are involved in the segregation of chromatids and are the result of the interaction of a myriad of proteins dictating the dynamics of microtubule nucleation through polymerization of α and β tubulin dimers. As shown in Figure 6, control and dasatinib treated cells exhibit normal mitotic spindle structures. Contrarily, treatment with crizotinib and combination for 48 h severely disrupts the formation of the mitotic spindle in LN-18 cells. This disruption is likely a consequence of the inhibition of aurora kinases by crizotinib and provides a plausible mechanism for the previously observed polynucleation (Figure 3) and polyploidy (Figure 5).

### The combination of crizotinib and dasatinib prevents migration, invasion, HUVEC tube formation and tube-like formation of GBM cells

**Figure 7:**
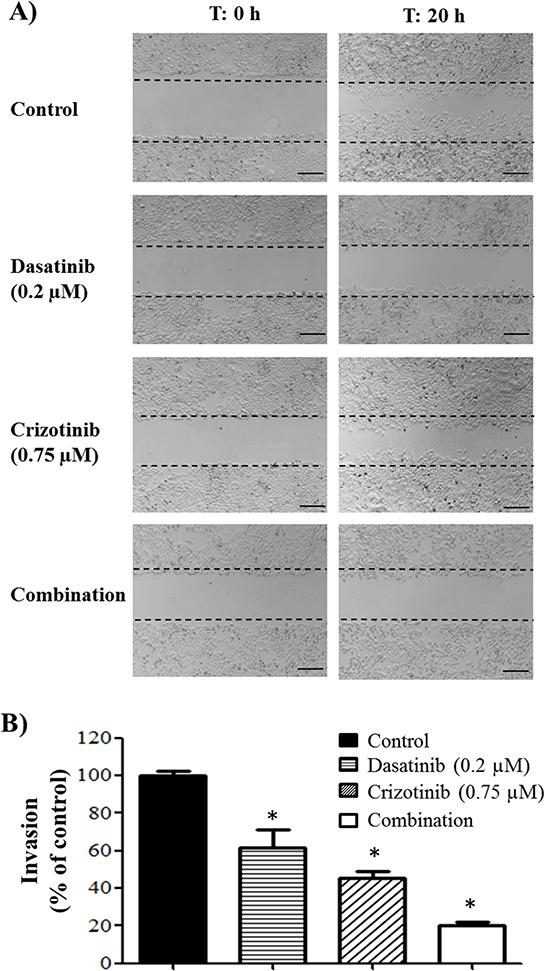
Migration and invasion of GBM cell lines **A.** Migration of LN-18 cells following 20 h incubation and treatment with dasatinib (0.2 μM) and/or crizotinib (0.75 μM). The experiments were conducted in triplicate and repeated independently three times, representative pictures are shown. The scale bar denotes 200 μm. **B.** Invasion of LN-18 cells through a Geltrex basement membrane following 24 h of incubation and treatment with dasatinib (0.2 μM) and/or crizotinib (0.75 μM). The experiments were conducted in triplicate and repeated independently three times, * denotes *p* ≤ 0.05 relative to control.

The capacity of GBM cells to invade the surrounding brain parenchyma is a serious impediment to the effective application of radiotherapy and chemotherapy. As seen in Figure 7A, migration of LN-18 cells is reduced following dasatinib and crizotinib treatment and abolished by combination treatment, at concentrations of 0.2 μM dasatinib and 0.75 μM crizotinib, insufficient to promote cell death after 20 h incubation. Furthermore, when the ability of cells to invade through a basement membrane was assessed, this sub-cytotoxic dose was sufficient to reduce the invasion of LN-18 cells by 80% following combination treatment (Figure 7B).

**Figure 8:**
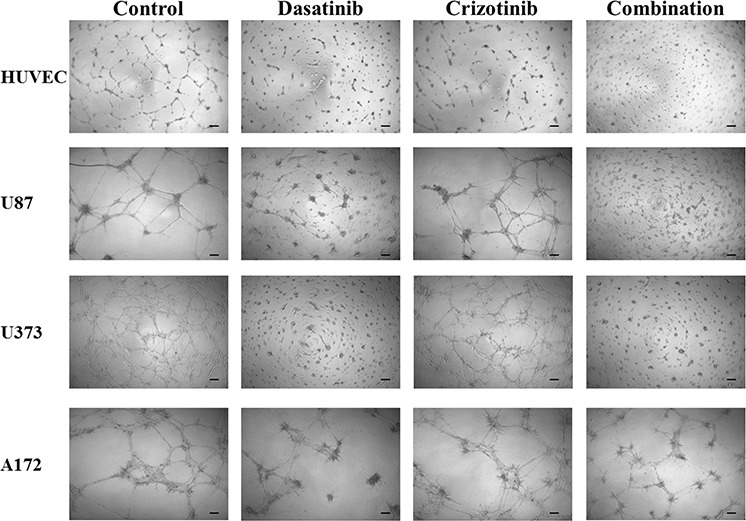
Tube formation of HUVEC cells and tube-like formation of U87, U373 and A172 cells Cells were seeded onto Geltrex matrix and treated for 20 h before representative were pictures taken. The scale bar denotes 100 μm.

*In vitro* models of angiogenesis, using HUVEC cells, and vascular mimicry, using tumor cells, have been employed to ascertain if the current drug combination shows potential efficacy. As can be seen in Figure 8, the tube-like formation of HUVEC is inhibited by treatment with dasatinib while treatment with crizotinib has a moderate effect. Following combination treatment there is no apparent tube-like formation of HUVEC cells. When U87, U373, and A172 cells are seeded onto the Geltrex basement membrane, they also form long projections resembling that of the HUVEC cells, termed vascular mimicry. In these cells combination treatment completely abolishes the formation of the tube-like projections in both U87 and U373 cells and greatly reduces tube-like formation in A172 cells. LN-18 cells do not form these projections and so were not included in this experiment.

## DISCUSSION

The development of alternative and effective ways to treat and reduce the recurrence of GBM is being actively explored. The Cancer Genome Atlas has identified the amplification of several RTKs such as EGFR, ERBB2, PDGFRα, Kit, vascular endothelial growth factor 2 (VEGFR-2), fibroblast growth factor receptor 2, insulin receptor substrate 2 and Met [24–26]. Tyrosine kinase inhibitors have emerged as effective cancer therapeutics in many types of cancers and are being assessed for the treatment of GBM. Currently, 139 clinical trials in phase I or II using 27 different TKIs targeting various receptor and non-receptor tyrosine kinases are active worldwide for the treatment of GBM. Although many molecular targets have already been identified, the efficacy of a specific TKI remains questionable due to the multiplicity, redundancy and heterogeneity of GBM signaling pathways which change during the course of the development of the tumor [27] and in response to treatment [28]. Furthermore, a subset of patients may have tumors with characteristics of more than one subtype [8]. Thus it is conceivable that the use of a combination of drugs would provide a better therapeutic outcome.

The present study described the response of four established and two primary GBM cell lines to a combination of the TKIs, crizotinib and dasatinib. Dasatinib inhibits multiple tyrosine kinases which are reported to be overexpressed or constitutively active in GBM such as the SRC family kinases, Kit, macrophage colony-stimulating factor receptor, PDGFR-α and–β, EphB1, EpHB2 and EphB4 [29–32]. The cross-examination of the broadly selective TKIs assessed in our study (Table 1) identified SRC as a protein of interest. SRC plays a key role in the signal transduction of a diverse panel of cell surface receptors such as Met, PDGFR, EGFR and EGFRvIII, expressed in 25–64% of GBM patients [33–38]. SRC is involved in the regulation of a variety of cellular processes including cell volume regulation, cell proliferation and invasion. In the cell lines considered in this study, SRC was constitutively active which has also been observed in patient tumor samples [12]. The other TKI used in the combination treatment, crizotinib, inhibits multiple tyrosine kinases including Met, RON and ALK [39]. In this study, we considered only the effect of treatment on Met expression due to the paucity of understanding of the contribution of ALK and ROS1 to GBM. Western blots confirmed that the signaling of both SRC and Met was suppressed by single and combination treatment while the activity of the proliferative and survival downstream effector AKT was effectively suppressed by the combination treatment in both established and primary cell lines. Furthermore, the combination treatment was sufficient to reduce the expression of tyrosine kinases known to be overexpressed and to contribute to the progression and invasion of GBM such as EGFR and FAK [40, 41].

Dasatinib has proven to be well tolerated in clinical trials, but failed to improve overall survival either as a monotherapy or when combined to erlotinib [42], lomustine [43] or bevacizumab [44] for recurrent GBM. The limited efficacy of dasatinib was attributed to poor accumulation in the brain due to the activity of ATP-binding cassette transporters such as P-glycoprotein (P-gp) and breast cancer resistance protein which are highly expressed on the blood-brain barrier (BBB) and glioblastoma cells [45, 46]. Our data and the data from previous studies [47] suggests that the limited efficacy may also be a consequence of the action of dasatinib being cytostatic as opposed to cytotoxic. This effect was highlighted by the absence of apoptosis and necrosis markers following treatment and the increase in G1 phase seen following 48 and 72 h dasatinib treatment (Figure 4).

No clinical trials examining the efficacy of crizotinib in GBM patients have been completed to date. Although, there have been several clinical trials initiated to test inhibitors of Met using small molecule inhibitors such as crizotinib (NCT02270034) and cabozantinib (NCT00704288) or antibodies directed against Met (NCT01632228). Crizotinib exhibits poor BBB penetration [48]; however, the BBB is often severely disrupted in the region of a tumor lesion and the pathological changes of the tumor vasculature increase permeability to small molecules [49]. Crizotinib is also a potent inhibitor of P-gp transport [41], it is possible that crizotinib treatment may prolonged the efficacy of a combination with dasatinib by reducing their efflux from the tumor tissue. Promisingly, combination treatment is sufficient to promote apoptotic cell death which was maintained for up to 72 hours following the removal of the drugs. This is an encouraging observation as it lowers the possibility of resistance emerging as the cells are committed to cell death.

The combination of dasatinib and crizotinib was shown to increase apoptosis but also to significantly reduce the proliferation of GBM cells. The promotion of cell cycle arrest in the G_2_/M phase of the cell cycle and the formation of polyploid cells by combination treatment was confirmed by western blot, FACS and immunocytochemistry analysis. Perturbations of the cell cycle were associated with a reduction of CD1 expression across all the cell lines. CD1 expression is generally associated with the transition from G_1_/S and S/G_2_, however in cancer cells, its expression is high throughout the S and G_2_/M phases, promoting the constitutive phosphorylation of the retinoblastoma protein [50, 51]. CD1 expression is regulated by SRC (Figure 2) and downregulation of SRC activity is sufficient to decrease CD1 expression and subsequently cell proliferation [52]. CD1 upregulation, however, was observed in crizotinib treated cells (Figure 2) and is associated with an increased proportion of polyploid cells [53], also observed following crizotinib treatment (Figure 5).

Cell cycle arrest in G_2_/M phase is usually sufficient to trigger apoptosis; in our study, the increased proportion of cells in the G_2_/M phase observed in the combination treatment is associated with a larger quantity of apoptotic cells. The G2/M phase accumulation can be the result of the formation of aberrant mitotic spindles observed following crizotinib and combination treatments (Figure 6) which is usually associated with the impaired segregation of chromosomes, G_2_/M arrest and increased apoptosis [54].

We also observed an increased number of polyploid (4X and 8X DNA) cells following crizotinib treatment which seems to be associated with a decreased number of apoptotic cells. Studies have suggested that polyploidy is associated with apoptosis resistance [55]. While the combination treatment also increased the proportion of polyploid cells relative to control the proportion of polyploid cells relative to crizotinib treatment was decreased. This combination may reduce treatment resistance by avoiding the genetic instability induced by crizotinib monotherapy.

Moreover, our results showed that the combination treatment decreased migration and reduced cellular invasion, both essential to metastases. The anti-invasive potential of the combination of dasatinib and crizotinib occurs at low crizotinib concentration. This effect is a particularly important observation as invasion into the surrounding healthy brain ultimately reduces the efficacy of all standard treatments for GBM. Traditional chemotherapies are often ineffective against invading cells as these cells suppress their proliferation, reducing the damage caused by agents that promote DNA damage [56]. Moreover, GBM lesions are highly vascularized [57] but antiangiogenic therapies show poor efficacy in GBM [58]. Our results show that the combination of dasatinib and crizotinib decreases angiogenesis and vascular mimicry in an *in vitro* model. The combination of the anti-angiogenic and anti-invasive properties is an essential consideration as the clinical usefulness of Bevacizumab, an antiangiogenic therapy, has been limited by the promotion of the invasive phenotype [59].

In conclusion, our results demonstrated that a combination of dasatinib and crizotinib was sufficient to promote apoptosis, reduce cell migration and invasion and abolish neo-angiogenesis. The combination was sufficient to reduce the activity of signaling pathways essential for GBM survival and treatment resistance. These findings suggest that the combination approach has therapeutic potential, and further studies are warranted to test its efficacy *in vivo*.

## MATERIALS AND METHODS

### Materials

Sorafenib, nilotinib, sunitinib, imatinib, gefitinib, lapatinib, dasatinib, PD-173074, selumetinib, crizotinib, tofacitinib and pazopanib were purchased from LC Laboratories (Woburn, Massachusetts, USA). Roswell Park Memorial Institute Medium (RPMI) and Geltrex basement membrane was purchased from Life Technologies (North Shore City, New Zealand). Met, P-Met (Y1234/Y1235), SRC, P-SRC (Y416), AKT, P-ATK (S473), cyclin D1, cleaved caspase 3, α-tubulin and EGFR antibodies were purchased from Cell Signaling Technology (Danvers, Massachusetts, USA). β-Tubulin was purchased from Sigma-Aldrich (Auckland, New Zealand).

### Cell culture

A172 and LN-18 cells were purchased from the American Type Culture Collection. U373 cells were generously provided by Dr. Andrew Bahn (University of Otago, New Zealand). Cells were cultured in RPMI medium supplemented with 5% fetal bovine serum (FBS) and maintained in a humidified atmosphere at 37°C, 5% CO_2_. Spheroids were grown in 96 well plates with a non-adherent base of 50 μL 1.5% agarose. Primary GBM cells (NZG0906 and NZG1003) were isolated and cultured from GBM material obtained from patients undergoing debulking surgery as described previously [60].

### Cytotoxicity

Cells were seeded in 96-well plates (U87: 6,000, LN-18: 5,000, U373: 4,000, A172: 4,000 cells/well) and incubated for 24 h. The cells were then treated as indicated in each experiment and incubated for a further 72 h. The cytotoxicity was assessed using a sulforhodamine B (SRB) assay as described previously [61]. Viability of spheroids was assessed using the acid phosphatase assay. Spheroids were collected into tubes, centrifuged for 5 minutes at 1000 RPM and washed 3 times with cold phosphate buffered saline (PBS). The supernatant was aspirated and 100 μL of cold PBS was placed on top and the spheroid sonicated. The solution was transferred to a 96 well plate and 100 μL of 0.1 M sodium acetate, 0.1% (vol/vol) Triton X-100 and 2 mg/mL para-Nitrophenylphosphate was added to each well. The solution was incubated at 37°C for 90 minutes before the reaction was halted using 10 μL of NaOH and the absorbance read at 405 nm deducting background at 630 nm. Acid phosphatase samples were conducted in sextuplicate and the experiment independently repeated 3 times.

### Proliferation

Spheroids were collected after treatment for 4 days and frozen in Optimum Cutting Temperature from Newcomer supply (Middleton, WI, USA). The spheroids were sectioned at 10 μm and incubated overnight with rabbit anti-Ki67 (Epitomics, Burlingame, CA, USA). Slides were incubated with biotinylated goat anti-rabbit (Dako, Campbellfield, Australia) and then with streptavidin (BD Pharmingen) before development with 3, 3′-diaminobenzidine tetrahydrochloride (DAB) (BD Pharmingen) and counterstaining with hematoxylin QS (Vector Laboratories).

### Mode of cell death

Cells were seeded in 6-well plates (65,000 cells/well) and incubated for 24 h. Cells were treated as indicated and incubated for 48 and 72 h. For washout experiments, the media was aspirated, and the cells washed twice with PBS, before addition of fresh growth media. At the end of the treatment period, apoptosis was assessed using Annexin-V-FLUOS/propidium iodide (PI) staining, as described previously [62]. Samples were analyzed using a FACScalibur flow cytometer, and the proportion of apoptotic cells was determined using CellQuest Pro software (BD Biosciences, San Jose, CA, USA).

### Cell cycle

Cells were seeded in a 6-well plate (65,000 cells/well) and incubated for 24 h before treatment. At the end of the treatment period, cell cycle distribution was assessed using PI staining, as previously described [62]. Cells were analyzed using a FACScalibur flow cytometer (BD Biosciences, San Jose, CA, USA) and the proportion of cells in each of G0/G1-, S- and G2/M-phases were determined using CellQuest Pro software.

### Immunoblotting

Cells were seeded in petri dishes (200,000 cells) and incubated for 24 h before being treated for 48 h. Following the treatment period, the cells were washed with PBS and lysed in Tris-HCl 50 mM (pH 8), sodium chloride (NaCl) 150 mM, Triton X-100 1%, sodium dodecyl sulfate (SDS) 1%, sodium fluoride (NaF) 1 mM, sodium orthovanadate 200 μM, and protease inhibitors (leupeptin 1 μg/mL, aprotinin 1 μg/mL, phenylmethylsulfonyl fluoride (PMSF) 1 mM). The lysates were cleared from insoluble material by centrifugation at 12,000 rpm for 10 min, subjected to SDS-polyacrylamide gel electrophoresis, and analyzed by western blotting.

### Indirect immunofluorescence microscopy

Cells were seeded onto a glass coverslip (20,000 cells/well) and incubated for 24 h before being treated for 48 h. The cells were washed with PBS and fixed with 4% paraformaldehyde overnight at 4°C. The cells were washed with Tris-buffered saline (TBS) twice and permeabilized with TBS: Triton X-100 0.2% for 5 min, washed with TBS and blocked with 1.5% goat serum. Cells were incubated with 60 μL of 50 μg/mL anti-α-tubulin, anti-Met or anti-SRC antibody overnight at 4°C, washed with TBS before incubation a secondary FITC or Texas red-labeled antibodies for 1 h at room temperature. Cells were washed with TBS before being incubated in 50 μL of 50 ng/mL 4′,6-diamidino-2-phenylindole (DAPI) in the dark for 30 minutes. Cells were washed with TBS and glass coverslips were mounted onto microscope slides using ProLong Gold antifade reagent.

### Migration

LN-18 cells were seeded in 6-well plates (100,000 cells/well) and grown to 80% confluence. Scratches were made in the cell monolayer followed by extensive washing to remove cell debris. Then growth media was added to the cells and treatments were applied. Representative photographs were taken, and the cells were incubated for 20 h before photographs were taken again.

### Invasion

LN-18 (20,000 cells) were seeded onto Geltrex (diluted in a 1:1 ratio with RPMI) coated invasion membranes (8 μm pore; BD Biosciences) with or without treatment for 20 h. Lower chambers contained RPMI supplemented with chemoattractant, 5% FBS. Cells from each well were counted under an inverted microscope at 200 × magnification. Data were collected from three independent experiments, each done in triplicate.

### Tube formation assay

Tube formation was carried out using human umbilical vein endothelial cells (HUVEC) as described by [62]. Briefly, HUVEC (1.5 × 10^4^ cells/well), U87 (1.5 × 10^4^ cells/well), U373 (1.4 × 10^4^ cells/well) and A172 (1.4 × 10^4^ cells/well) were seeded on the top of Geltrex layer in 96-well plates. Cells were seeded, treated as specified and incubated for 20 h at 37°C in 5% CO2 atmosphere. After the incubation time, pictures were taken at 20 × magnification.

### Statistics

Statistical analysis was carried out using GraphPad Prism™. In order to correct for the correlation, covariance and non-normal distribution of the compositional cell cycle data, the data was subjected to Logit transformation and analyzed using a two-way ANOVA with a Bonferroni post hoc test. All other data was assessed via a one-way ANOVA with a Bonferroni post-hoc test. Significance was set at *p* ≤ 0.05.

## SUPPLEMENTARY TABLES


