# Supplementary material for: A combination of tyrosine kinase inhibitors, crizotinib and dasatinib for the treatment of glioblastoma multiforme
Source: Oncotarget. 2015 Oct 16;6(35):37948–64. doi: 10.18632/oncotarget.5698 (PMC4741976; doi:10.18632/oncotarget.5698)
Supplement: Supplementary file 1 [file oncotarget-06-37948-s001.pdf]

SUPPLEMENTARY TABLES

Supplementary Table S1: Statistical analysis of U87 cell cycle

	48 hours		72 hours		24 hour washout		48 hour washout		72 hour washout	
1 X	Treatment	*P* (≤)	Treatment	*P* (≤)	Treatment	*P* (≤)	Treatment	*P* (≤)	Treatment	*P* (≤)
	Control V Dasatinib	NS	Control V Dasatinib	NS	Control V Dasatinib	NS	Control V Dasatinib	NS	Control V Dasatinib	NS
	Control V Crizotinib	0.001	Control V Crizotinib	0.001	Control V Crizotinib	0.001	Control V Crizotinib	0.001	Control V Crizotinib	0.001
	Control V Combo	0.001	Control V Combo	0.001	Control V Combo	0.001	Control V Combo	0.01	Control V Combo	0.001
	Source of variation					*P* value				
	Drug					≤0.0001				
	Time					≤0.0001				
	Interaction					≤0.0001				
S	48 hours		72 hours		24 hour washout		48 hour washout		72 hour washout	
	Treatment	*P* (≤)	Treatment	*P* (≤)	Treatment	*P* (≤)	Treatment	*P* (≤)	Treatment	*P* (≤)
	Control V Dasatinib	NS	Control V Dasatinib	NS	Control V Dasatinib	NS	Control V Dasatinib	NS	Control V Dasatinib	NS
	Control V Crizotinib	0.001	Control V Crizotinib	0.01	Control V Crizotinib	NS	Control V Crizotinib	0.001	Control V Crizotinib	NS
	Control V Combo	0.01	Control V Combo	0.05	Control V Combo	NS	Control V Combo	0.01	Control V Combo	NS
	Source of variation					*P* value				
	Drug					≤0.0001				
	Time					≤0.0001				
	Interaction					NS (0.23)				
2 X	48 hours		72 hours		24 hour washout		48 hour washout		72 hour washout	
	Treatment	P (≤)	Treatment	P (≤)	Treatment	P (≤)	Treatment	P (≤)	Treatment	P (≤)
	Control V Dasatinib	NS	Control V Dasatinib	NS	Control V Dasatinib	NS	Control V Dasatinib	NS	Control V Dasatinib	NS
	Control V Crizotinib	0.001	Control V Crizotinib	0.001	Control V Crizotinib	NS	Control V Crizotinib	NS	Control V Crizotinib	0.05
	Control V Combo	0.001	Control V Combo	0.001	Control V Combo	NS	Control V Combo	NS	Control V Combo	NS
	Source of variation					*P* value				
	Drug					NS (0.057)				
	Time					≤0.001				
	Interaction					≤0.0001				

(*Continued*)

	48 hours		72 hours		24 hour washout		48 hour washout		72 hour washout	
	Treatment	*P* (≤)	Treatment	*P* (≤)	Treatment	*P* (≤)	Treatment	*P* (≤)	Treatment	*P* (≤)
T	Control V Dasatinib	NS	Control V Dasatinib	NS	Control V Dasatinib	NS	Control V Dasatinib	NS	Control V Dasatinib	NS
	Control V Crizotinib	NS	Control V Crizotinib	NS	Control V Crizotinib	NS	Control V Crizotinib	0.01	Control V Crizotinib	NS
	Control V Combo	NS	Control V Combo	NS	Control V Combo	NS	Control V Combo	0.01	Control V Combo	0.01
	Source of variation					*P* value				
	Drug					≤0.0001				
	Time					≤0.0001				
	Interaction					≤0.0001				
4 X	48 hours		72 hours		24 hour washout		48 hour washout		72 hour washout	
	Treatment	*P* (≤)	Treatment	*P* (≤)	Treatment	*P* (≤)	Treatment	*P* (≤)	Treatment	*P* (≤)
	Control V Dasatinib	NS	Control V Dasatinib	NS	Control V Dasatinib	NS	Control V Dasatinib	NS	Control V Dasatinib	NS
	Control V Crizotinib	0.001	Control V Crizotinib	0.001	Control V Crizotinib	0.001	Control V Crizotinib	0.001	Control V Crizotinib	0.001
	Control V Combo	0.05	Control V Combo	0.001	Control V Combo	0.01	Control V Combo	0.001	Control V Combo	0.001
	Source of variation					*P* value				
	Drug					≤0.0001				
	Time					≤0.0001				
	Interaction					≤0.0001				
T	48 hours		72 hours		24 hour washout		48 hour washout		72 hour washout	
	Treatment	*P* (≤)	Treatment	*P* (≤)	Treatment	*P* (≤)	Treatment	*P* (≤)	Treatment	*P* (≤)
	Control V Dasatinib	NS	Control V Dasatinib	NS	Control V Dasatinib	NS	Control V Dasatinib	NS	Control V Dasatinib	NS
	Control V Crizotinib	NS	Control V Crizotinib	0.05	Control V Crizotinib	0.001	Control V Crizotinib	0.01	Control V Crizotinib	0.001
	Control V Combo	NS	Control V Combo	NS	Control V Combo	0.001	Control V Combo	NS	Control V Combo	NS
	Source of variation					*P* value				
	Drug					≤0.0001				
	Time					≤0.0001				
	Interaction					≤0.05				

(*Continued*)

	48 hours		72 hours		24 hour washout		48 hour washout		72 hour washout	
8 X	Treatment	*P* (≤)	Treatment	*P* (≤)	Treatment	*P* (≤)	Treatment	*P* (≤)	Treatment	*P* (≤)
	Control V Dasatinib	NS	Control V Dasatinib	NS	Control V Dasatinib	NS	Control V Dasatinib	NS	Control V Dasatinib	NS
	Control V Crizotinib	NS	Control V Crizotinib	NS0	Control V Crizotinib	0.001	Control V Crizotinib	0.001	Control V Crizotinib	0.001
	Control V Combo	NS	Control V Combo	NS	Control V Combination	0.001	Control V Combo	0.001	Control V Combo	0.001
	Source of variation					*P* value				
	Drug					≤ 0.0001				
	Time					≤ 0.0001				
	Interaction					≤ 0.0001				

X denotes the quantity of DNA in the G1 phase of the cell cycle. 2X corresponds to the G2M phase while 4X and 8X to tetraploid and octoploid cells. S denotes DNA synthesis phase and T denotes transitionary phase between the diploid, tetraploid and octoploid phases. Combo denotes combination and NS non-significant.

Supplementary Table S2: Statistical analysis of LN-18 cell cycle

	48 hours		72 hours		24 hour washout		48 hour washout		72 hour washout	
1 X	Treatment	*P* (≤)	Treatment	*P* (≤)	Treatment	*P* (≤)	Treatment	*P* (≤)	Treatment	*P* (≤)
	Control V Dasatinib	NS	Control V Dasatinib	NS	Control V Dasatinib	NS	Control V Dasatinib	NS	Control V Dasatinib	NS
	Control V Crizotinib	0.001	Control V Crizotinib	0.001	Control V Crizotinib	0.001	Control V Crizotinib	0.001	Control V Crizotinib	0.001
	Control V Combo	0.001	Control V Combo	0.001	Control V Combo	0.001	Control V Combo	0.001	Control V Combo	0.001
	Source of variation					*P* value				
	Drug					≤0.0001				
	Time					≤0.0001				
	Interaction					NS (0.082)				
S	48 hours		72 hours		24 hour washout		48 hour washout		72 hour washout	
	Treatment	*P* (≤)	Treatment	*P* (≤)	Treatment	*P* (≤)	Treatment	*P* (≤)	Treatment	*P* (≤)
	Control V Dasatinib	NS	Control V Dasatinib	NS	Control V Dasatinib	NS	Control V Dasatinib	NS	Control V Dasatinib	NS
	Control V Crizotinib	0.01	Control V Crizotinib	NS	Control V Crizotinib	NS	Control V Crizotinib	0.05	Control V Crizotinib	NS
	Control V Combo	NS	Control V Combo	NS	Control V Combo	NS	Control V Combo	NS	Control V Combo	NS
	Source of variation					*P* value				
	Drug					≤0.0001				
	Time					NS (0.053)				
	Interaction					≤0.0001				

(*Continued*)

	48 hours		72 hours		24 hour washout		48 hour washout		72 hour washout	
2 X	Treatment	*P* (≤)	Treatment	*P* (≤)	Treatment	*P* (≤)	Treatment	*P* (≤)	Treatment	*P* (≤)
	Control V Dasatinib	NS	Control V Dasatinib	NS	Control V Dasatinib	NS	Control V Dasatinib	NS	Control V Dasatinib	NS
	Control V Crizotinib	0.001	Control V Crizotinib	0.01	Control V Crizotinib	NS	Control V Crizotinib	NS	Control V Crizotinib	NS
	Control V Combo	0.001	Control V Combo	0.01	Control V Combo	NS	Control V Combo	NS	Control V Combo	NS
	Source of variation					*P* value				
	Drug					≤0.0001				
	Time					≤0.0001				
	Interaction					≤0.0001				
T	48 hours		72 hours		24 hour washout		48 hour washout		72 hour washout	
	Treatment	*P* (≤)	Treatment	*P* (≤)	Treatment	*P* (≤)	Treatment	*P* (≤)	Treatment	*P* (≤)
	Control V Dasatinib	NS	Control V Dasatinib	NS	Control V Dasatinib	NS	Control V Dasatinib	NS	Control V Dasatinib	NS
	Control V Crizotinib	NS	Control V Crizotinib	NS	Control V Crizotinib	NS	Control V Crizotinib	NS	Control V Crizotinib	NS
	Control V Combo	NS	Control V Combo	NS	Control V Combo	NS	Control V Combo	0.05	Control V Combo	0.05
	Source of variation					*P* value				
	Drug					≤0.0001				
	Time					≤0.0001				
	Interaction					NS (0.097)				
4 X	48 hours		72 hours		24 hour washout		48 hour washout		72 hour washout	
	Treatment	*P* (≤)	Treatment	*P* (≤)	Treatment	*P* (≤)	Treatment	*P* (≤)	Treatment	*P* (≤)
	Control V Dasatinib	NS	Control V Dasatinib	NS	Control V Dasatinib	NS	Control V Dasatinib	NS	Control V Dasatinib	NS
	Control V Crizotinib	0.001	Control V Crizotinib	0.001	Control V Crizotinib	0.001	Control V Crizotinib	0.001	Control V Crizotinib	0.001
	Control V Combo	0.001	Control V Combo	0.001	Control V Combo	0.001	Control V Combo	0.001	Control V Combo	0.001
	Source of variation					*P* value				
	Drug					≤0.0001				
	Time					≤0.0001				
	Interaction					≤0.0001				

(*Continued*)

	48 hours		72 hours		24 hour washout		48 hour washout		72 hour washout	
T	Treatment	*P* (≤)	Treatment	*P* (≤)	Treatment	*P* (≤)	Treatment	*P* (≤)	Treatment	*P* (≤)
	Control V Dasatinib	NS	Control V Dasatinib	NS	Control V Dasatinib	NS	Control V Dasatinib	NS	Control V Dasatinib	NS
	Control V Crizotinib	0.001	Control V Crizotinib	0.001	Control V Crizotinib	0.001	Control V Crizotinib	0.001	Control V Crizotinib	0.001
	Control V Combo	NS	Control V Combo	NS	Control V Combo	NS	Control V Combo	0.001	Control V Combo	0.001
	Source of variation					*P* value				
	Drug					≤0.0001				
	Time					≤0.0001				
	Interaction					≤0.0001				
8X	48 hours		72 hours		24 hour washout		48 hour washout		72 hour washout	
	Treatment	*P* (≤)	Treatment	*P* (≤)	Treatment	*P* (≤)	Treatment	*P* (≤)	Treatment	*P* (≤)
	Control V Dasatinib	NS	Control V Dasatinib	NS	Control V Dasatinib	NS	Control V Dasatinib	NS	Control V Dasatinib	NS
	Control V Crizotinib	NS	Control V Crizotinib	NS	Control V Crizotinib	0.001	Control V Crizotinib	0.001	Control V Crizotinib	0.001
	Control V Combo	NS	Control V Combo	NS	Control V Combo	0.001	Control V Combo	0.001	Control V Combo	0.001
	Source of variation					*P* value				
	Drug					≤0.0001				
	Time					NS (0.56)				
	Interaction					≤0.001				

X denotes the quantity of DNA in the G1 phase of the cell cycle. 2X corresponds to the G2M phase while 4X and 8X to tetraploid and octoploid cells. S denotes DNA synthesis phase and T denotes transitionary phase between the diploid, tetraploid and octoploid phases. Combo denotes combination and NS non-significant.

Supplementary Table S3: Statistical analysis of U373 cell cycle

1 X	48 hours		72 hours		24 hour washout		48 hour washout		72 hour washout	
	Treatment	*P* (≤)	Treatment	*P* (≤)	Treatment	*P* (≤)	Treatment	*P* (≤)	Treatment	*P* (≤)
	Control V Dasatinib	NS	Control V Dasatinib	NS	Control V Dasatinib	NS	Control V Dasatinib	NS	Control V Dasatinib	NS
	Control V Crizotinib	0.001	Control V Crizotinib	0.001	Control V Crizotinib	0.001	Control V Crizotinib	0.001	Control V Crizotinib	0.001
	Control V Combo	0.001	Control V Combo	0.001	Control V Combo	0.001	Control V Combo	0.001	Control V Combo	0.001
	Source of variation					*P* value				
	Drug					≤0.0001				
	Time					≤0.0001				
	Interaction					≤0.0001				

S	48 hours		72 hours		24 hour washout		48 hour washout		72 hour washout	
	Treatment	*P* (≤)	Treatment	*P* (≤)	Treatment	*P* (≤)	Treatment	*P* (≤)	Treatment	*P* (≤)
	Control V Dasatinib	NS	Control V Dasatinib	NS	Control V Dasatinib	0.05	Control V Dasatinib	NS	Control V Dasatinib	0.001
	Control V Crizotinib	0.001	Control V Crizotinib	0.001	Control V Crizotinib	0.001	Control V Crizotinib	NS	Control V Crizotinib	NS
	Control V Combo	NS	Control V Combo	NS	Control V Combo	NS	Control V Combo	NS	Control V Combo	0.01
	Source of variation					*P* value				
	Drug					≤0.0001				
	Time					≤0.0001				
	Interaction					≤0.0001				

2 X	48 hours		72 hours		24 hour washout		48 hour washout		72 hour washout	
	Treatment	*P* (≤)	Treatment	*P* (≤)	Treatment	*P* (≤)	Treatment	*P* (≤)	Treatment	*P* (≤)
	Control V Dasatinib	NS	Control V Dasatinib	NS	Control V Dasatinib	NS	Control V Dasatinib	NS	Control V Dasatinib	NS
	Control V Crizotinib	NS	Control V Crizotinib	NS	Control V Crizotinib	NS	Control V Crizotinib	NS	Control V Crizotinib	NS
	Control V Combo	0.001	Control V Combo	0.001	Control V Combo	0.05	Control V Combo	NS	Control V Combo	NS
	Source of variation					*P* value				
	Drug					≤0.0001				
	Time					≤0.0001				
	Interaction					≤0.0001				

(*Continued*)

	48 hours		72 hours		24 hour washout		48 hour washout		72 hour washout	
T	Treatment	*P* (≤)	Treatment	*P* (≤)	Treatment	*P* (≤)	Treatment	*P* (≤)	Treatment	*P* (≤)
	Control V Dasatinib	NS	Control V Dasatinib	NS	Control V Dasatinib	NS	Control V Dasatinib	NS	Control V Dasatinib	NS
	Control V Crizotinib	NS	Control V Crizotinib	NS	Control V Crizotinib	0.05	Control V Crizotinib	0.001	Control V Crizotinib	0.05
	Control V Combo	NS	Control V Combo	NS	Control V Combo	0.05	Control V Combo	0.001	Control V Combo	NS
	Source of variation					*P* value				
	Drug					≤0.0001				
	Time					≤0.0001				
	Interaction					≤0.0001				
4 X	48 hours		72 hours		24 hour washout		48 hour washout		72 hour washout	
	Treatment	*P* (≤)	Treatment	*P* (≤)	Treatment	*P* (≤)	Treatment	*P* (≤)	Treatment	*P* (≤)
	Control V Dasatinib	NS	Control V Dasatinib	NS	Control V Dasatinib	NS	Control V Dasatinib	NS	Control V Dasatinib	NS
	Control V Crizotinib	0.001	Control V Crizotinib	0.001	Control V Crizotinib	0.001	Control V Crizotinib	0.001	Control V Crizotinib	0.001
	Control V Combo	0.01	Control V Combo	0.01	Control V Combo	0.001	Control V Combo	0.001	Control V Combo	0.001
	Source of variation					*P* value				
	Drug					≤0.0001				
	Time					≤0.05				
	Interaction					≤0.0001				
T	48 hours		72 hours		24 hour washout		48 hour washout		72 hour washout	
	Treatment	*P* (≤)	Treatment	*P* (≤)	Treatment	*P* (≤)	Treatment	*P* (≤)	Treatment	*P* (≤)
	Control V Dasatinib	NS	Control V Dasatinib	NS	Control V Dasatinib	NS	Control V Dasatinib	NS	Control V Dasatinib	NS
	Control V Crizotinib	0.01	Control V Crizotinib	0.01	Control V Crizotinib	0.001	Control V Crizotinib	0.05	Control V Crizotinib	0.001
	Control V Combo	NS	Control V Combo	NS	Control V Combo	NS	Control V Combo	NS	Control V Combo	0.001
	Source of variation					*P* value				
	Drug					≤0.0001				
	Time					≤0.005				
	Interaction					≤0.05				

(*Continued*)

	48 hours		72 hours		24 hour washout		48 hour washout		72 hour washout	
	Treatment	*P* (≤)	Treatment	*P* (≤)	Treatment	*P* (≤)	Treatment	*P* (≤)	Treatment	*P* (≤)
8X	Control V Dasatinib	NS	Control V Dasatinib	NS	Control V Dasatinib	NS	Control V Dasatinib	NS	Control V Dasatinib	NS
	Control V Crizotinib	0.001	Control V Crizotinib	0.001	Control V Crizotinib	0.001	Control V Crizotinib	0.01	Control V Crizotinib	0.001
	Control V Combo	0.01	Control V Combo	0.01	Control V Combo	0.001	Control V Combo	0.001	Control V Combo	0.001
	Source of variation					*P* value				
	Drug					≤0.0001				
	Time					≤0.0001				
	Interaction					≤0.01				

X denotes the quantity of DNA in the G1 phase of the cell cycle. 2X corresponds to the G2M phase while 4X and 8X to tetraploid and octoploid cells. S denotes DNA synthesis phase and T denotes transitionary phase between the diploid, tetraploid and octoploid phases. Combo denotes combination and NS non-significant.

Supplementary Table S4: Statistical analysis of A172 cell cycle

	48 hours		72 hours		24 hour washout		48 hour washout		72 hour washout	
	Treatment	*P* (≤)	Treatment	*P* (≤)	Treatment	*P* (≤)	Treatment	*P* (≤)	Treatment	*P* (≤)
1X	Control V Dasatinib	NS	Control V Dasatinib	NS	Control V Dasatinib	NS	Control V Dasatinib	NS	Control V Dasatinib	NS
	Control V Crizotinib	0.001	Control V Crizotinib	0.001	Control V Crizotinib	0.001	Control V Crizotinib	0.001	Control V Crizotinib	0.001
	Control V Combo	0.001	Control V Combo	0.001	Control V Combo	NS	Control V Combo	0.001	Control V Combo	0.001
	Source of variation					*P* value				
	Drug					≤0.0001				
	Time					≤0.01				
	Interaction					≤0.0001				
S	48 hours		72 hours		24 hour washout		48 hour washout		72 hour washout	
	Treatment	*P* (≤)	Treatment	*P* (≤)	Treatment	*P* (≤)	Treatment	*P* (≤)	Treatment	*P* (≤)
	Control V Dasatinib	0.01	Control V Dasatinib	NS	Control V Dasatinib	NS	Control V Dasatinib	NS	Control V Dasatinib	NS
	Control V Crizotinib	NS	Control V Crizotinib	0.001	Control V Crizotinib	NS	Control V Crizotinib	NS	Control V Crizotinib	NS
	Control V Combo	NS	Control V Combo	0.001	Control V Combo	0.001	Control V Combo	0.01	Control V Combo	NS
	Source of variation					*P* value				
	Drug					≤0.01				
	Time					≤0.0001				
	Interaction					≤0.0001				

(*Continued*)

	48 hours		72 hours		24 hour washout		48 hour washout		72 hour washout	
2 X	Treatment	*P* (≤)	Treatment	*P* (≤)	Treatment	*P* (≤)	Treatment	*P* (≤)	Treatment	*P* (≤)
	Control V Dasatinib	NS	Control V Dasatinib	NS	Control V Dasatinib	NS	Control V Dasatinib	NS	Control V Dasatinib	NS
	Control V Crizotinib	0.001	Control V Crizotinib	0.001	Control V Crizotinib	0.001	Control V Crizotinib	0.001	Control V Crizotinib	0.001
	Control V Combo	0.001	Control V Combo	0.001	Control V Combo	NS	Control V Combio	0.001	Control V Combo	0.05
	Source of variation					*P* value				
	Drug					≤0.0001				
	Time					≤0.0001				
	Interaction					≤0.0001				
T	48 hours		72 hours		24 hour washout		48 hour washout		72 hour washout	
	Treatment	*P* (≤)	Treatment	*P* (≤)	Treatment	*P* (≤)	Treatment	*P* (≤)	Treatment	*P* (≤)
	Control V Dasatinib	NS	Control V Dasatinib	NS	Control V Dasatinib	NS	Control V Dasatinib	NS	Control V Dasatinib	NS
	Control V Crizotinib	NS	Control V Crizotinib	NS	Control V Crizotinib	NS	Control V Crizotinib	NS	Control V Crizotinib	NS
	Control V Combo	NS	Control V Combo	NS	Control V Combo	NS	Control V Combo	NS	Control V Combo	NS
	Source of variation					*P* value				
	Drug					≤0.05				
	Time					≤0.0001				
	Interaction					NS (0.91)				
4 X	48 hours		72 hours		24 hour washout		48 hour washout		72 hour washout	
	Treatment	*P* (≤)	Treatment	*P* (≤)	Treatment	*P* (≤)	Treatment	*P* (≤)	Treatment	*P* (≤)
	Control V Dasatinib	NS	Control V Dasatinib	NS	Control V Dasatinib	NS	Control V Dasatinib	NS	Control V Dasatinib	NS
	Control V Crizotinib	NS	Control V Crizotinib	0.001	Control V Crizotinib	0.01	Control V Crizotinib	0.001	Control V Crizotinib	0.001
	Control V Combo	NS	Control V Combo	NS	Control V Combo	NS	Control V Combo	0.01	Control V Combo	0.01
	Source of variation					*P* value				
	Drug					≤0.0001				
	Time					NS (0.108)				
	Interaction					≤0.01				

(*Continued*)

	48 hours		72 hours		24 hour washout		48 hour washout		72 hour washout	
T	Treatment	*P* (≤)	Treatment	*P* (≤)	Treatment	*P* (≤)	Treatment	*P* (≤)	Treatment	*P* (≤)
	Control V Dasatinib	NS	Control V Dasatinib	NS	Control V Dasatinib	NS	Control V Dasatinib	NS	Control V Dasatinib	NS
	Control V Crizotinib	0.05	Control V Crizotinib	NS	Control V Crizotinib	NS	Control V Crizotinib	NS	Control V Crizotinib	NS
	Control V Combo	0.05	Control V Combo	NS	Control V Combo	NS	Control V Combo	NS	Control V Combo	NS
	Source of variation					*P* value				
	Drug					NS (0.065)				
	Time					≤0.0001				
	Interaction					NS (0.36)				
8X	48 hours		72 hours		24 hour washout		48 hour washout		72 hour washout	
	Treatment	*P* (≤)	Treatment	*P* (≤)	Treatment	*P* (≤)	Treatment	*P* (≤)	Treatment	*P* (≤)
	Control V Dasatinib	NS	Control V Dasatinib	NS	Control V Dasatinib	NS	Control V Dasatinib	NS	Control V Dasatinib	NS
	Control V Crizotinib	NS	Control V Crizotinib	NS	Control V Crizotinib	NS	Control V Crizotinib	NS	Control V Crizotinib	NS
	Control V Combo	NS	Control V Combo	NS	Control V Combo	NS	Control V Combo	NS	Control V Combo	NS
	Source of variation					*P* value				
	Drug					NS (0.22)				
	Time					≤0.01				
	Interaction					NS (0.64)				

X denotes the quantity of DNA in the G1 phase of the cell cycle. 2X corresponds to the G2M phase while 4X and 8X to tetraploid and octoploid cells. S denotes DNA synthesis phase and T denotes transitionary phase between the diploid, tetraploid and octoploid phases. Combo denotes combination and NS non-significant.
